# Development and Optimization of a Novel Lozenge Containing a Metronidazole-Peppermint Oil-Tranexamic Acid Self-Nanoemulsified Delivery System to Be Used after Dental Extraction: In Vitro Evaluation and In Vivo Appraisal

**DOI:** 10.3390/pharmaceutics15092342

**Published:** 2023-09-19

**Authors:** Mohammed Alissa, Ahmed Hjazi, Ghadah S. Abusalim, Ghfren S. Aloraini, Suad A. Alghamdi, Waleed Y. Rizg, Khaled M. Hosny, Jazia A. Alblowi, Hanaa Alkharobi

**Affiliations:** 1 Department of Medical Laboratory Sciences, College of Applied Medical Sciences, Prince Sattam bin Abdulaziz University, Al-Kharj 11942, Saudi Arabia; a.hijazi@psau.edu.sa (A.H.); g.abusalim@psau.edu.sa (G.S.A.); g.aloraini@psau.edu.sa (G.S.A.); s.alghamdi@psau.edu.sa (S.A.A.); 2 Department of Pharmaceutics, Faculty of Pharmacy, King Abdulaziz University, Jeddah 21589, Saudi Arabia; wrizq@kau.edu.sa (W.Y.R.); kmhomar@kau.edu.sa (K.M.H.); 3 Center of Innovation in Personalized Medicine (CIPM), 3D Bioprinting Unit, King Abdulaziz University, Jeddah 21589, Saudi Arabia; 4 Department of Pharmaceutics and Industrial Pharmacy, Faculty of Pharmacy, Beni-Suef University, Beni-Suef 62511, Egypt; 5 Department of Periodontology, Faculty of Dentistry, King Abdulaziz University, Jeddah 21589, Saudi Arabia; jaalblowi@kau.edu.sa; 6 Department of Oral Biology, Faculty of Dentistry, King Abdulaziz University, Jeddah 21589, Saudi Arabia; halkharobi@kau.edu.sa

**Keywords:** metronidazole, peppermint oil, tranexamic acid, nanoemulsion, hemostatic, optimization, lozenges, tooth extraction, periodontitis

## Abstract

In-depth studies on essential oil–based nanoemulsions (NEs) have centered on a variety of oral health issues. NEs improve the delivery of nonpolar active agents to sites and thereby boost the dissolution and distribution of the agents. Metronidazole-peppermint oil-tranexamic acid self-nanoemulsifying drug delivery systems (MZ-PO-TX-SNEDDS) were created and loaded into novel lozenges to act as antifungal, hemostatic, antimicrobial, and analgesic dosage forms after dental extractions. The design-of-experiments approach was used in creating them. To generate the NEs, different concentrations of MZ-PO (240, 180, and 120 mg), 2% TX (600, 450, and 300 mg), and S_mix1:1_ (600, 400, and 200 mg) were used. The ideal formulation had serum levels of 1530 U/mL of interleukin-6, a minimal inhibitory concentration against bacteria of 1.5 µg/mL, a droplet size of 96 nm, and a blood coagulation time of 16.5 min. Moreover, the produced NE offered better MZ release. The adopted design was used to produce the ideal formulation; it contained 240 mg of MZ-PO, 600 mg of 2% TX, and 600 mg of S_mix1:1._ It was incorporated into lozenges with acceptable characteristics and an improved capability for drug release. These lozenges had reasonable coagulation times, IL-6 serum levels, and MIC values. All of these characteristics are desirable for managing symptoms following tooth extractions. Therefore, these lozenges loaded with MZ-PO-TX-SNEDDs might be considered a beneficial paradigm for relieving complications encountered after tooth extractions.

## 1. Introduction

Tooth extraction is one of the most frequently carried out surgical procedures, and it is usually done by general dentists [1,2]. Despite the consistent downturn in numbers of regular extractions of permanent teeth recorded in the past few decades [3] and the steep decrease in the frequency of severe tooth loss [4], dentists still might remove up to seven teeth weekly. Patients in their sixth and seventh decades of life have the highest percentage of tooth extractions per patient [5]. Caries and periodontal ailments are the primary reasons for permanent tooth extraction. Following extraction, complications can be dangerous and occasionally lethal [6]. Such complications can result from situations such as the failure to administer local anesthesia, failure to pull out the tooth, breaking of the tooth or root, breaking of the alveolus, damaging the soft tissues around the tooth, bleeding, breaking of the mandible, damaging the nerve, and infection of the soft tissues [7]. Patients should be given good instructions on how to handle and control the complications that might arise after surgical treatment [8]. Dentists now frequently utilize antibiotics and antifungals for the management of odontogenic infections following tooth extractions [9]. In addition, several analgesics are employed following the surgical removal of impacted molars to treat postoperative pain and edema. Nonsteroidal anti-inflammatory drugs and paracetamol are frequently prescribed; they are regarded as standard medications by many medical professionals and might be used with corticosteroids or opioids [10].

One of the popular solid dosage forms is the lozenge, which conveys drugs to the mouth or pharynx. Lozenges have various benefits. They may be administered to people who have trouble swallowing [11]; they increase the time an active agent spends in the oral cavity to produce a particular impact; they have a pleasing flavor; and they are simple to deliver to both infants and elderly people [12]. The administration of lozenges does not require medical intervention, as the administration of parenterals does, and patients do not need to take them with water. Lozenges are simple to develop, and little time and equipment are needed for their production [13]. One of the most often utilized local hemostatic medications for dental operations is tranexamic acid (TX), an antiplasmin drug that aids in achieving hemostasis by blocking the proteolytic breakdown of fibrin [14]. A study has shown that patients who received warfarin along with 4.8% TX mouthwash after dental procedures had a lower incidence of postoperative hemorrhage than patients who took warfarin and did not receive TX. This demonstrated the effectiveness of local TX treatment [15].

One of the cornerstone drugs for the handling of protozoal, anaerobic bacterial, and microaerophilic bacterial infections is metronidazole (MZ) [16]. It enters microorganisms via dissemination, interacts with DNA to hinder protein production, destroys DNA strands, and compromises the composition of helical DNA, eventually destroying bacterial cells [17]. Due to its ease of procuration, its pharmacokinetics and pharmacodynamics, its manageable profile of unwanted side effects, and its unabated antimicrobial influence, MZ is a cost-effective treatment [18]. Newer, more expensive combination therapies have diminished MZ’s importance in managing combined aerobic/anaerobic infections, but they have not been demonstrated to offer any therapeutic advantages over MZ [19].

**Figure 1:**
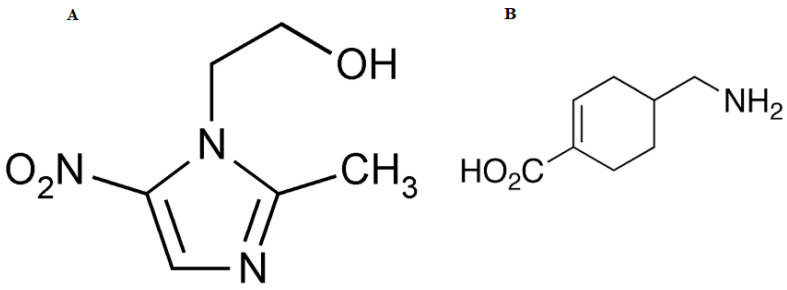
Chemical structure of metronidazole (**A**) and tranexamic acid (**B**).

After normal dental extractions, MZ has been proven to be successful in preventing the disorder known as “dry socket”, which may be linked to oral anaerobic bacteria [20]. Previous research has proven that 3% of regular tooth extractions result in dry sockets, and that they occur almost exclusively in the mandible [21]. However, controlling infections due to anaerobic organisms may be crucial in preventing dry sockets or resolving them quickly. It has been demonstrated that the prophylactic administration of MZ is an easy and efficient form of prophylaxis, and this implies that anaerobic organisms are involved in dry sockets [22]. Figure 1 illustrates the chemical structures of MZ and TX molecules.

Peppermint oil (PO; menthol) is one of the botanicals with the strongest antibacterial, antifungal, and antiviral properties, according to numerous investigations [23]. It is also efficient in the management of both obligate and facultative anaerobes [24]. Additionally, it functions as an antimicrobial and is resistant to at least 20 common enteric infections, caused by organisms such as *Shigella flexneri*, *Escherichia coli*, and *Staphylococcus aureus* [25,26,27,28]. Studies also showed that the menthol in PO has anti-inflammatory properties [29]. Menthol inhibits the in vitro synthesis of inflammatory mediators by human monocytes [30]. Transient receptor potential cation channel subfamily M member 8 (TRPM8) is also present in immune cells. Due to the fact that activating TRPM8 in mice models reduces chemically induced colitis, it is thought that PO’s anti-inflammatory properties may be partially mediated by this protein [31,32].

Colloidal particle systems in the nanosize range known as nanoemulsions (NEs) have been found to serve as efficient vehicles for medicinal molecules; they range from 10 to 1000 nm in size [33]. As drug delivery systems, NEs have earned praise for their strength. They can serve as a replacement for nanovesicles such as liposomes [34]. They may increase the absorption of active agents because of their large surface area, which they have gained owing to their extremely small droplet size [35]. They are usually nontoxic and nonirritating. They are known to enhance physical stability. They can be made in many different dosage forms, including foams, creams, liquids, and sprays. In cell culture techniques, they offer a greater uptake of oil-soluble supplements [36]. They aid in solubilizing lipophilic medications and, hence, they are considered useful for concealing unpalatable tastes [37].

Traditionally, all drug delivery methodologies have been developed using a trial-and-error technique that involved changing one variable at a time, but reaching an ideal formulation was not possible. Researchers can provide a suitable outline for creating the most optimized delivery system thanks to the systematic design-of-experiments (DoE) strategy [38]. In order to find the best answer to a specific issue, optimization methods utilizing DoE help to provide efficient and affordable analytical tools. For instance, screening designs involve many screening factors in a small number of tests, and it is advantageous to minimize the variables in a required size, making it simple to conduct additional experiments utilizing these variables [39]. The techniques that are methodically applied to classify different types of issues that could affect the invention of active agents’ delivery systems include experimental designs and optimization techniques [40]. The best method for screening and optimizing experimental variables is to use experimental design.

The goal of this research was to develop a novel lozenge loaded with an MZ-PO-TX-NE that could be used in an antifungal, hemostatic, antimicrobial, and analgesic dosage form after dental extractions, applying the DoE approach.

## 2. Materials and Methods

### 2.1. Materials

The Amoun Company provided TX (El-Obour City, Cairo, Egypt). The MZ was a kind present from Bayer (Leverkusen, Germany). Tedia Corporation provided the following products: gelatin, hydroxypropyl cellulose, propylene glycol, oleic acid, carbopol, and PO (Fairfield, OH, USA). Gattefosse kindly donated Labrafac Lipophile WL 1349, polyglyceryl oleate, and Transcutol Plurol (Saint-Priest, France). Sigma-Aldrich provided Tween 80, Span 80, polyethylene glycol (PEG) 200, PEG 400, propylene glycol, glycerol, and ethanol (St. Louis, MO, USA). All additional chemicals and reagents were of normal analytical grades.

### 2.2. Methodology

#### 2.2.1. Estimation of the Required Hydrophilic-Lipophilic Balance (RHLB) for MZ-Loaded PO

For PO in water, the reported RHLB is 12.3 [41]. Discrete proportions (i.e., 0.532:0.468, 0.579:0.421, 0.626:0.374, 0.672:0.328, 0.719:0.281, 0.766:0.234, 0.813:0.187, 0.859:0.141, 0.906:0.094) of Tween 80: Span 80 were employed to attain the best RHLB of the surfactant mixture (fluctuating between 10 and 14) needed to distribute PO loaded with 25% MZ in 2% aqueous TX solution.

In order to create the organic phase, 0.2 g of PO that had been loaded with MZ was vortexed with 0.4 g of a surfactant mixture. The nonpolar substance was then poured dropwise into a polar phase that contained 2% TX in distilled water, and the mixture was again vortexed. The ideal HLB of a surfactant mixture that would create an MZ-loaded PO emulsion in 2% aqueous TX solution was found by measuring the droplet size of the formed emulsions with Zetatrac (Microtrac, Montgomeryville, PA, USA) [42].

#### 2.2.2. Solubility Studies

The solubility of MZ in a number of cosurfactants, including glycerin, ethyl alcohol, ethanol, Transcutol, propylene glycol, PEG 200, and PEG 400, was examined. Excess amounts of the MZ powder being tested were added to vials holding 3 mL of each cosurfactant. After being safely stoppered, these vials were maintained for 3 days at a temperature of 25 ± 0.5 °C in an isothermal shaking water bath. After that, methanol was used to dilute the samples’ supernatant, which was obtained after centrifuging the samples at 4000 rpm for 30 min. Then, the methanol solution was filtered through a membrane of 0.45 µm. A UV-visible spectrophotometer was used to measure the solubility of MZ at a maximum of 277 nm, and the measurement was carried out three times [43].

#### 2.2.3. Construction of Pseudoternary Phase Diagrams

The pseudoternary phase graphs included the oil phase (MZ-loaded PO), the surfactant/cosurfactant combination (S_mix_) of Tween 80/Span 80 with an HLB of 11 and PEG 200, and the 2% TX aqueous solution. The MZ-PO, S_mix_, and 2% TX water solution were combined to form the NEs. The amphiphilic blend was used in various ratios (i.e., 1:1, 1:2, 1:3, 2:1, and 3:1). Different weight ratios of MZ-PO and S_mix_ were combined in accordance with each phase diagram, and these combinations were progressively diluted with the water phase (2% TX solution) until the total weight of the mixture reached 1 g. The NE area for each evaluated S_mix_ was then calculated by measuring the transparency of the mixtures. This was carried out not by heating the mixture but by using moderate magnetic stirring [44].

#### 2.2.4. Statistical Designing of Experiments and Optimization of Self-Nanoemulsifying Drug-Delivery Systems (SNEDDS)

A 3D response surface design (i.e., Box–Behnken design) was used to determine the impacts of the studied factors on the in vitro attributes and in vivo fruitfulness metrics for the developed self-nanoemulsifying drug delivery systems (SNEDDS). The design was established using Design Expert software (version 12.0.6.0; Stat-Ease, Inc., Minneapolis, MN, USA). The examined factors were the quantities of MZ-PO in milligrams (A), amounts of 2% TX aqueous solution in milligrams (B), and amounts of S_mix1:1_ in milligrams (C). The investigated dependent responses included the droplet size of the NE (Y_1_), coagulation time (Y_2_), amounts of interleukin-6 (IL-6) (Y_3_), and minimal inhibitory concentrations (MICs) against *Treponema denticola* (Y_4_). Table 1 shows the independent variables and their levels, along with the chosen dependent responses, according to the chosen design.

#### 2.2.5. MZ-PO-TX–Loaded NEs

The MZ-loaded NEs were developed in two separate steps. The initial step involved the development of drug-free SNEDDS. Following the guidelines of the design, calculated amounts of the S_mix1:1_ were combined with a specific quantity of 25% MZ-loaded PO. The final NE was created in the second step by diluting the simple SNEDDS with a predetermined quantity of aqueous 2% TX solution using a vortex. As shown in Table 2, the quantities of each component were used in accordance with those of the design.

#### 2.2.6. Characterization of the NEs

##### Droplet Size (Y_1_)

The droplet sizes of the prepared NEs were measured by blending 100 µL of each MZ-PO-TX-NE with 900 µL of refined water in a volumetric flask. After the formulation had been vigorously mixed, 100 µL of the prepared sample was used to ascertain the droplet size and polydispersity index (PDI) using a Zetatrac particle size analyzer (Microtrac, Inc., Montgomeryville, PA, USA) [33].

##### Blood Coagulation Time (Y2)

Animal Handling and Care

The animals were treated and cared for in compliance with the guidelines established by the Animal Ethics Committee of the Cairo Agriculture for Experimental Animals, Cairo, Egypt, Approval No (115-11-22). Researchers followed the guidelines of the Declaration of ARRIVE and its Guiding Principles for the Care and Use of Animals. The laboratory-based investigational male albino rats with average weight oscillating from 235 to 20 gm were housed in cages and had unrestricted access to standard rat chow pellets and water ad libitum. The rats received the appropriate care in order to minimize their agony. The animals were given 14 days at least to adjust before the trial began. The trial used realistic conditions at an ambient temperature, 55.5% relative humidity, and a 12 h cycle of light and darkness. Forty-five adult rats were used in the trial, and they were split up into 15 groups of 3 rats each.

The in vivo periodontitis model used the following procedure. To put the rodents to sleep, 10% chloral hydrate was intraperitoneally injected. They were then placed in the supine position and their mouths were fully opened using a handmade mouth expander. The gums were completely separated, exposing the bilateral maxillary first teeth, using a gingival separator. For a span of 2 weeks, an orthodontic steel filament (0.2 mm in diameter) was used to tie each rat’s two maxillary first teeth together. The ligature did not impede the rats’ capacity to eat or impact their buccal mucosa because the bonding wires were positioned in the gingival sulcus. The periodontitis model was created in some groups in 1 day. To check the control coagulation time, a blood sample (1 mL) was first drawn from the orbital venous plexus of one animal from each group before treatment with the nanoformulations to be tested. Then, each MZ-PO-TX nanoemulsion was introduced into the buccal, lingual, and mesial gingiva of the maxillary molars in addition to the adjoining tooth space found amidst the maxillary molars.

Estimation of Coagulation Time (Y_2_)

Four tubes were used for each of the formulations evaluated in the coagulation time assay. One tube was used as the reference, and three more were used for the test; the results of the three tubes were averaged and there were three measurements. The control tube held 0.1 mL of NaCl 0.9%, and each of the three tubes used for the test contained 0.1 mL of the NE to be tested. The donor’s venous blood was then drawn, yielding 0.2 mL, and the timing was measured by a stopwatch as soon as the blood entered the syringe. The tubes were instantly put in a water bath (Fisher Scientific Polytest 20, Illkirch, France), which was set at 37 °C and sealed with carded cotton. Five minutes later, the tubes were tilted at a 45-degree angle for 60 s intervals to see if the blood had fully coagulated. When the tube could be turned around without significantly moving the blood inside, coagulation was deemed to be complete. Each tube’s blood coagulation time was noted, along with the average coagulation time for the triplicates of each examined sample [45].

##### IL-6 Level (Y_3_)

Blood samples from the animals previously treated with the NEs were used for this test. The amount of IL-6 was measured using the quantitative sandwich ELISA (R&D Systems, Inc., Minneapolis, MN, USA). A monoclonal antibody specific to rat IL-6 had been pre-coated on a microplate, and it was associated with any rat IL-6 found in the sample. After eliminating any unbound substances, a rat IL-6-specific enzyme-coupled polyclonal antibody was introduced; the blue color that developed turned to yellow, and the intensity of the color was evaluated to determine the concentration of IL-6 in each of the test specimens [46].

##### Antibacterial Activity (Y4)

A complicated polymicrobial infection called periodontal disease is marked by tooth loss, gingival inflammation, and alveolar bone resorption. Several hundred different bacterial species, including 60 different anaerobic treponeme species, may make up the subgingival microflora, or spirochetes, in the periodontal cavity. Periodontal ailments have been closely associated with a subset of oral bacteria, of which *Treponema denticola* is one. Each sample had its MIC values determined using the dilution technique. Each evaluated NE was diluted in a stock solution (0.5 mg/mL in PBS) and equilibrated in anaerobic conditions.

The MIC determination method that involves broth dilution was adopted. *T. denticola* (2 × 10^6^ cells/mL) was inoculated in MIC assays with escalating amounts of the tested NE analogues from 0.0 to 10.0 g/mL in 10 mL of media (pre-equilibrated in an anaerobic chamber). Over the course of 7 days, aliquots of 10 L were removed every 24 h, and cell counts were calculated using dark-field microscopy. We counted and averaged the number of *T. denticola* in three different fields of vision. The MIC was ascertained to be the lowest NE concentration at which growth was suppressed after 72 h. Three duplicates of each trial were carried out [47].

#### 2.2.7. Optimization of the MZ-PO-TX-NE

The *F*-ratio, *p*-value, and degrees of freedom for each assessed element and their interlinkages were calculated for the ANOVA testing of the models. Based on the outcomes, the model that matched the obtained data best was selected. *p*-values lower than 0.05 especially demonstrated the significance of the model parameters. The coefficient of variance (CV%) values, determination factors’ values, predicted R-squared values, and adjusted R-squared values were used to further appraise the model’s appropriateness. The best variables were selected based on the hypothesis that the generated MZ-PO-TX-NEs could meet four goals, namely, reduce the droplet size, coagulation time, amounts of IL-6, and MIC values.

#### 2.2.8. Characterization of the Optimized MZ-PO-TX-NEs

The droplet size, coagulation time, amounts of IL-6, and MIC values of the optimized formulation were all quantified and contrasted with the theoretical values of the identical dependent factors recommended by the software. Next, each formulation’s thermodynamic stability was examined to determine how well it could be incorporated into a lozenge. The entrapment efficiency (EE%) and in vitro release behavior of the optimal formulation were also assessed.

##### Stability Index Determination

To demonstrate the thermodynamic stability of the optimized MZ-PO-TX-NE, an accelerated investigation of the freeze-thaw stability at various temperatures was carried out. The samples underwent three rounds of freezing (at −25 °C for roughly 24 h) and thawing (at +25 °C for the following 24 h) after the droplet size was determined. Each iteration in a row ended with a measurement of the droplet size. The equation below was used to determine the stability index [48]:

Stability index = ([Initial size − Change in size]/Initial size) × 100(1)

##### Evaluation of EE% of MZ in the Optimized MZ-PO-TX-NE

Calculations were made to determine how much MZ was contained in the optimized mixture. The mixtures were placed in vials, which were placed in a shaking water bath at 25 ± 2 °C for 24 h. After achieving balance, each NE’s supernatant was separated by centrifugation for roughly 15 min at 4500 rpm. After being gathered and carefully washed, the precipitates were dissolved in methanol. Using a UV-visible spectrophotometer, the medication was extracted and the quantity of MZ was measured and evaluated at a λ_max_ of 277 nm. The drug-entrapment effectiveness was calculated using the following formula [43]:

(2) $$\mathrm{EE}\left(\%\right)=\frac{C\;\mathrm{total}-\;C\;\mathrm{free}}{C\;\mathrm{total}}$$

where C_total_ was the theoretical added concentration and C_free_ was the drug concentration in the supernatant fluid.

##### In Vitro Release Study of MZ-PO-TX-NEs

According to a previously documented method [48], in vitro release studies were implemented using the Microette Plus Hanson Automated Vertical Diffusion Cell (Hanson Research, Chatsworth, CA, USA). Phosphate-buffered saline (PBS, pH 6.8) served as the receptor in the receptor chamber, which was kept at a temperature of 37 ± 0.5 °C with 400 rpm of stirring. A UV-visible spectrophotometer was used to measure the amounts of MZ that were released and penetrated the cellulose membrane, as well as to compare the amounts of MZ released from the optimal NE with those released from the aqueous drug suspension. The MZ determination was carried out at a λ_max_ of 277 nm.

#### 2.2.9. Formulation and Characterization of the Lozenge Loaded with Optimized MZ-PO-TX-NE

The lozenge containing the optimized MZ-PO-TX-NE was fabricated using a glycerin mixture that made up 10% of the gross weight of the lozenge’s base. Ten grams of the ideal MZ-PO-TX-NE were combined with 20 mL of glycerin. After being sonicated, this combination was kept in a water bath at 37 °C until it was added to the final preparation. Ten grams of dextrose had been fully dissolved in 20 mL of water, and the mixture was heated to 120 °C. The result was a transparent viscous syrup. Following that, 20 mL of sugar syrup was added to the dextrose solution, which was then heated to 160 °C until the color turned golden yellow. The temperature of the mixture was lowered to 80 °C, and then the mixture was further mixed with medicated glycerin. The mass was spread over a lubricated mold and permitted to cool to an ambient temperature [49].

#### 2.2.10. Characterization of Lozenges

By using industry-accepted pharmaceutical standards, the lozenges were assessed for in vitro disintegration, thickness and diameter, drug content, friability, hardness, and weight. Additionally, an in vitro drug dissolution study using the USP II paddle at 150 rpm was conducted in 250 mL of PBS with a pH of 6.8. The in vitro release behavior of MZ from the MZ aqueous dispersion was compared with its release behavior from lozenges containing the drug aqueous suspension or the optimal NE.

##### Stability Studies

An 8-week accelerated stability study was carried out in accordance with ICH recommendations (zone IV) at 45 °C and 75% relative humidity. An ample number of lozenges were placed in amber-colored bottles fitted with a screwcap and stored in an incubator that was kept at 37 °C. These lozenges were loaded with the optimized MZ-PO-TX-NEs. In order to determine the drug content and assess the samples’ hardness, friability, in vitro disintegration, and in vitro drug dissolution properties, sampling periods of 15 days were employed [13].

##### Assessment of MIC Values, IL-6 Levels, and Coagulation Times for the Optimized Lozenges Loaded with the Optimized MZ-PO-TX-NEs

The optimized lozenge was placed in 25 mL of PBS (pH 6.8) at 37 °C for 30 min in a water bath sonicator before being evaluated for its MIC value, amount of IL-6, and coagulation time. The same procedures as previously stated were used. For the coagulation time and IL-6 measurements, 2 groups of male albino rats each composed of three rodents were employed. The tests were carried out three times.

## 3. Results and Discussion

### 3.1. Calculation of RHLB for PO Loaded with MZ

As shown in Table 2, using a combination of Tween80 and Span80 at a ratio of 0.626:0.374 resulted in the smallest globule of the MZ-PO-TX-NE, which was 295 ± 18 nm. As a result, the RHLB for the MZ-PO in the TX aqueous solution was 11, since this was the RHLB that corresponded to the smallest droplet size. Therefore, Tween80/Span80 in a ratio of 0.626:0.374 was employed for additional research on creating the optimal MZ-PO-TX-NE.

### 3.2. Pseudoternary Phase Diagram

**Figure 2:**
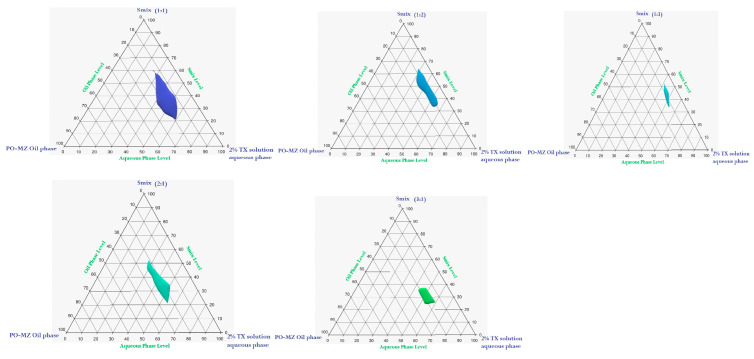
The pseudoternary phase diagram of the MZ-PO phase, 2% TX-aqueous phase, and S_mix_.

It was noteworthy that an investigation of the NE region showed that MZ-PO could be used at 12% to 25%, while the levels of S_mix_ varied from 30% to 60% and the 2% TX aqueous phase varied from 20% to 60%. Therefore, in order to create an NE, a combination of surfactant and cosurfactant (S_mix_) would need to be used in a ratio of 1:1 surfactant to cosurfactant as shown in Figure 2.

### 3.3. Solubility Study

**Figure 3:**
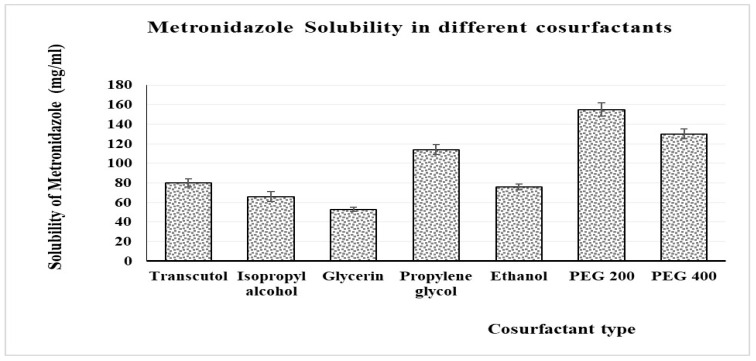
Measured solubility of MZ in different cosurfactants (means ± SD, *n* = 3).

Figure 3 shows that the greatest solubility of MZ was obtained with PEG 200 (156 ± 3 mg/mL), so this cosurfactant was chosen to be used in the formulation of the NEs as a component of the S_mix_.

### 3.4. Characterization of MZ-PO-TX-NEs

#### 3.4.1. Droplet Size

The PDI estimates of the NEs ranged from 0.12 to 0.36, and their droplet diameters ranged from 73 ± 2.1 to 181 ± 5.6 nm (Table 3). These figures demonstrated the formulations’ conformity, stability, and size dispersion.

The droplet size data of the fabricated NEs were evaluated by quadratic polynomial analysis. Based on the mathematical formula, the study model was reliable in finding the effect of the amount of MZ-PO (A), percentage of TX aqueous solution (B), and amount of S_mix1:1_ (C) on the droplet sizes of the MZ-PO-TX-NEs. The selected model had an adjusted R-squared value of 0.9967 and a predicted R-squared value of 0.9833, which were correlated, as shown in Table 4. The following algorithm was developed using an ANOVA data analysis:

(3) $$\mathrm{Droplet}\;\mathrm{szie}=+117+13\;A+1.37\;B-39.13\;C+0.5000\;\mathrm{AB}-1.5\;\mathrm{AC}-0.2500\;\mathrm{BC}+0.1250{\;A}^{2}-1.12{\;B}^{2}+8.37{\;C}^{2}$$

As can be seen from the above equation, factor A (amount of MZ-PO) and factor C (amount of S_mix1:1_) had a significant impact on the droplet size of the NEs (*p* < 0.0001).

Factor C (i.e., S_mix1:1_) exerted a negative effect on the studied response because it was able to lower the interfacial tension between the aqueous and nonaqueous phases and thereby create smaller droplets. The increase in the level of the surfactant–cosurfactant mixture was able to reduce the size of the oil droplets of the emulsion [46]. On the contrary, factor A (MZ-PO amount) had a positive impact on the measured droplet sizes. Thus, increasing the amount of factor A resulted in a comparable increase in the droplet size. Increasing the amount of PO might have enabled the formation of larger droplets by allowing more MZ, a lipophilic drug, to be encapsulated within droplets of the nonpolar phase of the NE [50]. The amount of 2% TX solution was found to have a nonsignificant impact on the studied response (*p* > 0.08).

**Figure 4:**
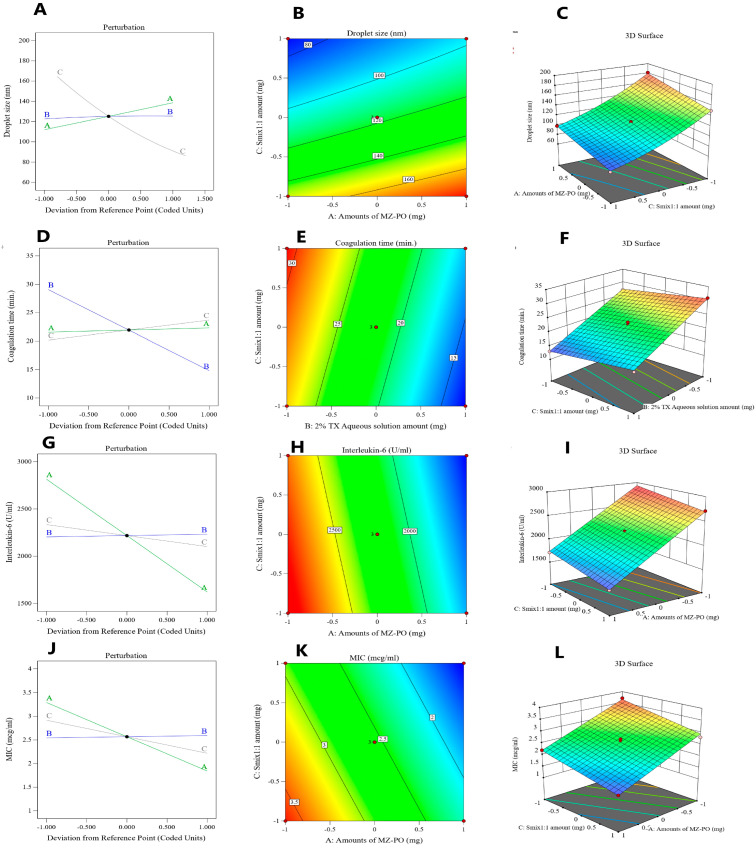
Effects of different independent elements on the droplet size of various MZ-PO-TX-NEs as shown by (**A**) Perturbation plot, (**B**) contour plot, and (**C**) 3D surface plot showing the; effects of different independent elements on the blood coagulation time in rats after administration of various MZ-PO-TX-NEs as presented by (**D**) Perturbation plot, (**E**) contour plot, and (**F**) 3D surface diagram; The impacts of various independent factors on IL-6 serum levels in rats following the administration of various MZ-PO-TX-NEs as shown in the (**G**) perturbation, (**H**) contour, and (**I**) 3D surface diagrams; The influences of various explored parameters on MIC values following the use of various MZ-PO-TX-NEs are shown in the (**J**) perturbation, (**K**) contour, and (**L**) 3D surface diagrams.

The size of the MZ-PO-TX-NE droplets was affected by the investigated factors, as shown by the perturbation, contour graphs, and 3D surface in Figure 4A, B, and C, respectively. The diagrams clarify how the amounts of MZ-PO and S_mix1:1_ impacted the size of the droplets.

#### 3.4.2. Blood Coagulation Time

The developed formulations’ various abilities to coagulate blood in all tested animals were demonstrated by the blood coagulation time values of the NEs, which varied from 13 ± 0.5 to 31 ± 2.2 min (Table 3). It was noted that the control tubes that were withdrawn from animals before treatment with the NEs acquired relatively longer coagulation times that ranged between 35 and 38 min.

A linear polynomial analysis model was applied to the collected blood coagulation time data of the NEs. The research model was successful in identifying the significant effects and limits of the amounts of MZ-PO (A), 2% TX aqueous solution (B), and S_mix1:1_ (C) on the MZ-PO-TX-NEs’ capacity to coagulate blood, in accordance with the chosen mathematical design. As shown in Table 4, the model had an adjusted R-squared value of 0.9907 and a predicted R-squared value of 0.9869, which were strongly correlated. The following formula was produced by performing an ANOVA data analysis:

(4) Coagulation time=+21.93+0.3750 A−7.13 B+1.75 C 

By carefully examining the equation, it can be noticed that factor A (amount of MZ-PO) did not have much of an impact on the blood coagulation time in the animals (*p* = 0.0743). Contrasted with these findings, factor B (2% TX aqueous solution) and factor C (amount of S_mix1:1_) had a significant impact on the studied response (*p* < 0.0001). The amount of 2% TX aqueous solution (factor B) had a negative significant influence on the blood coagulation time. Therefore, increasing the amount of TX aqueous solution resulted in a comparable decrease in the time required for blood to coagulate in animals treated with the NEs. Such findings showed the outstanding anticoagulating effect of TX. As a synthetic lysine derivative, TX inhibits plasminogen from interacting with newly developed plasmin and fibrin to produce antifibrinolytic effects by preventing lysine binding sites on plasminogen molecules [51]. As a consequence, the preformed fibrin meshwork of secondary hemostasis is stabilized when plasminogen activation is inhibited, and this eventually stops hemorrhaging [52].

On the other hand, factor C (amount of S_mix1:1_) had a significant positive impact on the blood coagulation time in the animals. This meant that by raising the amount of the surfactant mixture, the time required for blood to coagulate would also increase. These effects could be explained by employing previously reported outcomes. The in vitro experiments performed by Findaly et al. [53] demonstrated that cell membranes’ exposure to surfactants may increase membrane porosity and cause cytotoxicity, which may then result in the development of hemorrhage. Moreover, the induced cellular stretch may inhibit the production of the tissue factor procoagulant, which could reduce the production of thrombin, according to a prior theory [54]. Another study proved that surfactant interferes with blood coagulation by reducing platelet aggregation. This was shown by the Impact-R results in that study, which destroyed the whole blood platelet function, resulting in an increased tendency for bleeding [55].

The blood coagulation time in animals treated with the MZ-PO-TX-NEs was governed by the investigated parameters, as shown by the perturbation, contour, and 3D surface graphs in Figure 4D, E, and F, respectively. The diagrams illustrate how the amounts of 2% TX and S_mix1:1_ in the NEs impacted the studied response.

#### 3.4.3. Serum IL-6 Measurements

IL-6, a protein that assists in regulating immune responses, is commonly produced by cells. IL-6 is a helpful indicator of immune system activation and inflammation that is usually increased in response to microbial infection, inflammation, immune system disorders, and (rarely) cancer [56].

Rats receiving the fabricated NEs had IL-6 serum values of between 1500 ± 24 and 2862 ± 35 IU/mL. Referring to the experimental plan and the mean values of the IL-6 plasma limits, a linear model of polynomial formulas was generated to determine the impact of the explored factors on IL-6 serum levels. The model showed an adjusted R-squared value of 0.9945, which was very close to the predicted R-squared value of 0.9910, as seen in Table 4. The ANOVA evaluation of the data produced the formula below:

(5) Interleukin−6=+2218−596.50 A+14.12 B−116.13 C

The formula revealed that both factors A and C had a negative significant influence on IL-6 serum levels. Hence, the increase in both factors would lower the IL-6 levels, clarifying the anti-inflammatory effect of both variables.

Investigations have shown that the menthol in PO has anti-inflammatory properties [29]. Xylene-induced gastrointestinal inflammation in mice and acetic acid-induced colitis in rats were prevented by ingesting PO orally [29,57]. It was established that menthol inhibited the in vitro generation of inflammatory mediators by human monocytes [58]. Transient receptor potential cation channels, one of which is TRP melastatin 8 (TRPM8), are present in immune cells. Because the activation of TRPM8 in mouse models reduced chemically induced colitis, it is thought that the anti-inflammatory benefits of PO may be partially mediated by this protein [32,59]. Additionally, MZ was used to treat a number of noninfectious ailments, but its exact mode of action is still unknown. In a prior investigation of the anti-inflammatory properties of MZ, it was found that it inhibited leukocyte-endothelial attachment [60]. By altering neutrophil activity and lowering radical oxygen derivatives, MZ may have an anti-inflammatory effect, according to other research [61,62].

A smaller globule size was seen with higher amounts of S_mix1:1_, and this led to a larger surface area used for drug release and permeation, which would ultimately increase the effectiveness of the explored factors in lowering the IL-6 values. This may be the cause of the indirect correlation between the amounts of S_mix_ and the levels of IL-6 in plasma [46].

The perturbation, contour, and 3D surface graphs in Figure 4G, H and I, respectively demonstrate how the investigated factors influenced the IL-6 plasma limits in animals given MZ-PO-TX-NEs. These graphs show the considerable impact of the amounts of MZ-PO and S_mix1:1_ in the formulations on the investigated response.

#### 3.4.4. Antibacterial Activity Evaluation (Y_4_)

By quantifying the MIC values, it was possible to test the resistance of *T. denticola* to the NEs. The values of MIC varied between 1.5 ± 0.11 and 3.7 ± 0.23 µg/mL, as shown in Table 3.

A linear paradigm of polynomial formulas was created to evaluate the impact of the investigated independent variables on MIC values depending on the selected statistical plan and the estimated mean MIC values. The model’s adjusted R-squared value of 0.9799, as shown in Table 4, was very near the predicted R-squared value of 0.9699. The data collected from the ANOVA analysis led to the following equation:

(6) MIC=+2.57−0.7250 A+0.0250 B−0.3500 C 

As can be seen in the preceding formula, both factors A and C had a negative significant influence on the MIC values. Hence, an increase in both factors would lower the MIC values, affirming the significant antibacterial effect of both variables.

It was noticed that increasing factor A (amount of MZ-PO) decreased the MIC values of the tested formulations. These findings can be ascribed to the antibacterial activity of both PO and MZ. The major components of peppermint essential oil are menthol (38% to 48%), menthones (20% to 30%), monoterpens and oxides, and monoterpinic alcohols [63]. These constituents are known to work well as antibiotic, antiviral, and antiseptic agents. Menthol can damage organisms and may even give rise to the increased fluidization and subsequent infiltration of intracellular components, destabilizing microbial organisms, owing to its ability to exist in the aqueous extracellular medium and engage with the phospholipid membranes [64]. MZ, the other component of factor A, enters the targeted microorganism via permeation, interacts with DNA to hinder protein development, destroys DNA strands, and impairs the helical DNA structure. Hence, it leads to cell death in vulnerable organisms.

The MIC of *T. denticola* was substantially impacted negatively by the S_mix_ ratio used (*p* = 0.0001). The droplet size may be a factor in these findings. The droplet size decreased as the amount of S_mix_ increased. A larger surface area would be accessible for MZ release and permeation via the microbial cell wall due to the observed decrease in droplet size brought about by the increase in the amount of S_mix_. These actions would lead eventually to increased bacterial death and a reduction in the MIC of *T. denticola*. Additionally, the amphoteric nature of the used surfactant mixture may contribute to the antibacterial activity of factor C. Surfactants may be able to disrupt the bacterial cell membrane and hence increase the cell membrane fluidity and leakage of cellular components, resulting in bacterial death [46].

The perturbation, contour, and 3D surface graphs in Figure 4J, K and L, respectively demonstrate how the explored factors influenced the MIC values following treatment with the MZ-PO-TX-NEs. These graphs show the considerable impact of the MZ-PO and S_mix1:1_ amounts in the formulations on the investigated response. Table 4 reveals the ANOVA analysis of measured responses.

### 3.5. Optimization of the MZ-PO-TX-NEs

The ideal NE with the most suitable properties was found after conducting all the experiments described. For various combinations of levels of the variables under investigation, Design-Expert software version 13.0.12.0 suggested a number of solutions. The ideal formulation contained 600 mg of S_mix1:1_, 240 mg of MZ-PO, and 600 mg of 2% TX. The optimal formulation had a mean droplet size of 96 nm, an IL-6 value of 1530 U/mL, an MIC value against *T. denticola* of 3.7 µg/mL, and a blood coagulation time of 16.5 min with 0.874 desirability. Table 5 shows the adjusted and predicted values of the measured responses, and they appear to be closely related.

### 3.6. Characterization of the Optimized MZ-PO-TX-NE

#### 3.6.1. Stability Index Determination

The optimal NE had a stability index of 93%, which was an acceptable value and comparable to others found in the literature [48]. It signified the thermodynamic stability of the obtained formulation.

#### 3.6.2. Evaluation of the EE% of MZ in the Optimized MZ-PO-TX-NE

The optimal formulation had an EE% of MZ of 86%, which indicated the reasonable ability of the designed drug delivery system to carry the investigated drug. The lipophilic nature of MZ might have enabled it to be well incorporated within the nonaqueous phase of the optimal NE [65].

#### 3.6.3. In Vitro Release Study of MZ-PO-TX-NE

**Figure 5:**
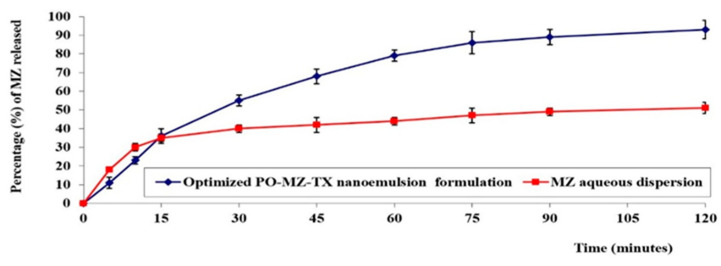
In vitro release profiles of MZ from optimal NE and MZ aqueous dispersion.

As shown in Figure 5, the percentage of MZ released after 120 min from the optimized MZ-PO-TX-NE reached 93 ± 4%, while the percentage released from the MZ aqueous dispersion was 49 ± 2%. The enhanced drug release observed for the optimal NE could be due to the S_mix_ content of the NE, which might have affected the drug release by boosting the dissolution of MZ. Another factor could have been the minute size of the fabricated nanodroplets of the emulsion, which could have made a larger surface area available for MZ release [66]. On the other hand, the low percentage of release in the MZ aqueous suspension might be ascribed to the poor water solubility of MZ.

### 3.7. Characterization of Lozenges

All the fabricated lozenges were found to be within the acceptable weight limits (193 to 207.5 mg). The average hardness and thickness of the lozenges were in the ranges of 11 ± 0.5 kg/cm^2^ and 3.100 ± 0.015 mm, respectively. The lozenges had an average diameter of 1.5 ± 0.05 cm, and the drug content was higher than 96% and the friability less than 2% for all formulations. The in vitro disintegration was higher than 25% within 3 min, 37% within 5 min, and 61% within 15 min, and complete disintegration occurred within 30 min. All the data indicated that the manufactured lozenges could be effectively used in drug delivery, and similar outcomes were reported in the literature [67].

#### 3.7.1. In Vitro Release Study

**Figure 6:**
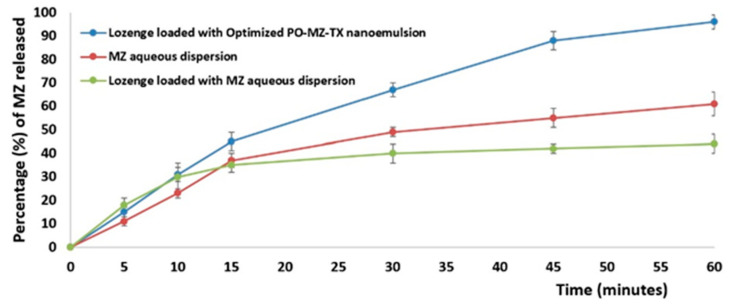
In vitro release of MZ from different lozenges and the drug aqueous suspension.

It could be observed from the Figure 6, that the lozenges containing the optimal NE had the highest release percentage (90 ± 2.2%) and that the lozenges containing the MZ aqueous dispersion had the lowest drug release percentage (40 ± 3.5%); the MZ aqueous dispersion had a drug release percentage of 60 ± 2.8%. The enhanced release of MZ from the optimum NE could be due to the amphiphilic molecules in the NE as well as the small size of the NE’s droplets [66].

Interestingly, the MZ released from the drug aqueous suspension was greater than that released from lozenges containing the MZ dispersion. This could be due to the expected hindrance of drug diffusion through the lozenge and the sugar coating around it.

#### 3.7.2. Stability Study

The prepared formulation showed excellent stability after 8 weeks of storage, and all tested parameters were within an acceptable range.

#### 3.7.3. Assessment of MIC Values, IL-6 Levels, and Coagulation Times for the Optimal Lozenge Loaded with the Optimal MZ-PO-TX-NE

The tested formulation had a blood coagulation time of 18 ± 1 min, an IL-6 serum level of 1580 ± 89 IU/mL, and an MIC value of 1.4 ± 0.1 µg/mL. There were some differences in the values of the tested parameters between the lozenges containing the optimal NE and the dispersion of the optimal NE. However, these differences were found to be insignificant (*p* < 0.05).

## 4. Conclusions

A functional NE was effectively fabricated by combining MZ, PO, and TX. A pseudoternary phase graph was employed to determine the best levels of S_mix_, oil phase, and TX that would generate the best drug delivery paradigm. The generated NEs were fairly stable because they had droplet diameters of less than 200 nm and an appropriate homogeneous distribution. Blood coagulation times of 13 ± 0.5 to 31 ± 2.2 min, MIC values of 1.5 ± 0.11 and 3.7 ± 0.23 g/mL, and IL-6 serum level values of 1500 ± 24 to 2862 ± 35 IU/mL were also obtained for the NEs. The response surface Box–Behnken design was used to produce the ideal NE, which contained 600 mg of S_mix1:1_, 240 mg of MZ-PO, and 600 mg of 2% TX. The ideal formulation had an EE% of 86%, a stability index of 93%, and a better release behavior of MZ compared with the drug dispersion. The best formulation was incorporated into lozenges, which exhibited reasonable quality control parameters in addition to an acceptable coagulation time, IL-6 serum levels, and MIC values. It is expected that in terms of patient compliance, quick start of action, extended retention period, improved bioavailability, ease of manufacture, localized drug targeting, and reduced dose frequency, the developed lozenges would offer a number of benefits and be capable of effectively treating symptoms following dental extractions. 

## Figures and Tables

**Table 1:** Box–Behnken design of NEs, showing the independent variables and their levels plus the dependent variables and their restrictions.

Studied Factors	Levels		
	1	0	−1
A = Amounts of MZ-PO (mg)	240	180	120
B = 2% TX Aqueous solution amount (mg)	600	450	300
C = S_mix1:1_ amount (mg)	600	400	200
Dependent variables	Constrains		
Y_1_ = Droplet size (nm)	Minimize		
Y_2_ = Coagulation time (minutes)	Minimize		
Y_3_ = Interleukin-6 (U/mL)	Minimize		
Y_4_ = minimum inhibitory concentration (MIC) against *Treponema denticola* (µg/mL)	Minimize		

**Table 2:** RHLB and globule sizes of MZ-PO-TX-NEs formulated using different ratios of Tween 80/Span 80.

RHLB	Tween 80 Ratio	Span 80 Ratio	Droplet Size of Formed PO-MZ-TX Emulsion
10	0.532	0.468	421 ± 38 nm
10.5	0.579	0.421	348 ± 24 nm
11	0.626	0.374	295 ± 18 nm
11.5	0.672	0.328	370 ± 16 nm
12	0.719	0.281	445 ± 37 nm
12.5	0.766	0.234	477 ± 29 nm
13	0.813	0.187	522 ± 36 nm
13.5	0.859	0.141	568 ± 40 nm
14	0.906	0.094	603 ± 41 nm

**Table 3:** Box–Behnken design of MZ-PO-TX-NEs’ compositions and the associated responses.

Run	A:Amounts of MZ-PO (mg)	B:2% TX Aqueous Solution Amount (mg)	C:S_mix1:1_ Amount (mg)	Y_1_:Droplet Size (nm)	Y_2_:Coagulation Time (min)	Y_3_:Interleukin-6 (U/mL)	Y_4_:MIC (µg/mL)	PDI
1	0	0	0	116 ±1.8	23 ± 0.2	2217 ± 50	2.7 ± 0.20	0.15
2	0	0	0	118 ± 3.1	22 ± 0.8	2241 ± 42	2.6 ± 0.16	0.12
3	1	−1	0	126 ± 3.5	29 ± 1.0	1630 ± 30	1.7 ± 0.15	0.25
4	0	−1	1	85 ± 1.1	31 ± 2.2	2033 ± 37	2.3 ± 0.22	0.31
5	−1	0	1	73 ± 2.1	24 ± 1.3	2713 ± 51	2.8 ± 0.14	0.19
6	0	0	0	117 ± 2.2	22 ± 0.4	2228 ± 38	2.6 ± 0.11	0.27
7	0	−1	−1	161 ± 7.0	27 ± 1.1	2339 ± 49	2.9 ± 0.10	0.33
8	1	1	0	130 ± 1.9	15 ± 0.5	1645 ± 19	1.8 ± 0.23	0.18
9	1	0	1	98 ± 0.5	24 ± 1.8	1500 ± 24	1.5 ± 0.11	0.36
10	−1	−1	0	103 ± 1.4	28 ± 2.2	2844 ± 41	3.2 ± 0.27	0.22
11	−1	0	−1	150 ± 5.9	20 ± 1.7	2862 ± 35	3.7 ± 0.23	0.28
12	−1	1	0	105 ± 2.5	14 ± 1.9	2839 ± 55	3.3 ± 0.18	0.32
13	0	1	−1	164 ± 2.7	13 ± 0.5	2369 ± 62	2.9 ± 0.09	0.16
14	1	0	−1	181 ± 5.6	21 ± 1.2	1711 ± 42	2.2 ± 0.13	0.29
15	0	1	1	87 ± 4.1	16 ± 3.1	2106 ± 45	2.3 ± 0.21	0.3

**Table 4:** ANOVA analysis for the selected models for different responses of MZ-PO-TX-NEs.

Dependent Variables	Model	R^2^	Adjusted R^2^	Predicted R^2^	Sequential *p*-Value	*F*-Value	Lack of Fit *p*-Value	Adequate Precision	Significant Terms	PRESS
Y_1_	Quadratic	0.9988	0.9967	0.9833	0.0001	475.03	0.0299	70.8239	A, C, C^2^	232
Y_2_	linear	0.9927	0.9907	0.9869	0.0001	497.30	0.6527	63.8954	B, C	5.68
Y_3_	linear	0.9957	0.9945	0.9910	0.0976	847.59	0.0976	80.9488	A, C	26,788.49
Y_4_	linear	0.9842	0.9799	0.9699	0.0001	228.36	0.3129	47.8345	A, C	0.1568

**Table 5:** Adjusted and predicted values of the best MZ-PO-TX-Nes.

Solution	PO-MZ Amount (mg)	S_mix1:1_ Amount (mg)	2% TX Amount (mg)	Droplet Size (nm)	Blood Coagulation Time (min)	MIC (μg/mL)	IL-6 Level (IU/mL)	Desirability
Expected value	240	600	600	98.3	16.9	1.51	1519	0.874
Tentative value	240	600	600	96	16.5	1.50	1530	0.874

## Data Availability

The data presented in this study are available on request from the corresponding author. The data are not publicly available as it was originally produced through research.
